# Effects of a stepwise, local patient-specific early oral feeding schedule after gastric cancer surgery: a single-center retrospective study from China

**DOI:** 10.1038/s41598-019-52629-0

**Published:** 2019-11-12

**Authors:** Ji Chen, Ming Xu, Yunpeng Zhang, Chun Gao, Peng Sun

**Affiliations:** Department of General Surgery, Tongren Hospital, Shanghai Jiao Tong University School of Medicine, 1111 Xianxia Road, Shanghai, 200336 China

**Keywords:** Gastric cancer, Nutrition, Surgical oncology

## Abstract

Nowadays, early oral feeding after gastrectomy has been gradually accepted and applied in the clinical practice, but there is still no specific uniform feeding regimen available which works best for patients in different regions with different races and eating habits. Aiming to establish an early oral feeding schedule suitable for local Chinese patients after gastric surgery, from May 2014 to May 2018, 87 gastric cancer patients undergoing various types of gastric resections were enrolled in an early feeding protocol and their clinical course was reviewed retrospectively. A stepwise, local patient-specific, early oral feeding schedule was proposed, implemented within an early recovery after surgery (ERAS) protocol and accessed in terms of its safety and tolerability. The primary surgical outcomes included: a median (interquartile range; IQR) postoperative hospital stay of 6 (3) days; 67 (77%) patients were well tolerant of this schedule from postoperative day (POD) 1 to POD 4; 20 (23%) patients had mild I/II grade complications (Clavien-Dindo classification); 3 (3%) patients had IIIB complications, zero cases of hospital mortality. Compared to similar studies in the past, our early oral feeding program is also safe and beneficial, and it can shorten the postoperative hospital stay without causing any increase in postoperative complications. In summary, our work herein reported the establishment of a detailed early oral feeding schedule embedded within an ERAS protocol which was found to be suitable for local Chinese patients after gastric surgery. Accordingly, this early oral feeding schedule is worth further research and promotion.

## Introduction

Gastric cancer is one of the most frequent malignant carcinomas in the world^1–3^. In recent years, there are about 680,000 new cases in China each year, accounting for about half of the global cases, and most of them are diagnosed at advanced stages, where resection is the only proven treatment to prolong the survival of gastric cancer patients^4^. In order to avoid postoperative complications of anastomotic leakage and postoperative paralytic ileus, the traditional idea is that feeding should not be started for patients after gastric resection until flatus or defecation has documented the return of bowel function^5,6^.

However, multiple lines of evidence demonstrate that early postoperative oral feeding has a good effect and has been considered as a safe and feasible method for patient management after colorectal cancer resection^7–12^. As one of the most important elements of multimodal Enhanced Recovery After Surgery (ERAS) protocols, which was first proposed by Kehlet in the late 1990s^13,14^, early postoperative oral feeding, often referred to as early feeding within 48 hours after surgery, has been more and more adopted in clinical practice^7,12,15–17^. Over the past 20 years, the ERAS program has been widely implemented in colorectal surgery and has been shown to significantly reduce postoperative stress response and positively affect short-term outcomes^18–20^. Despite this, the situation in gastric cancer surgery has not been well accepted^6^. This is mainly due to the fact that patients undergoing gastrectomy surgery are less tolerant to oral feeding than patients undergoing colorectal surgery, and therefore ERAS treatment for gastric resection is not as attractive for colorectal surgery.

The purpose of this single-center retrospective study was to establish a well-tolerated early oral feeding regimen for our local gastric cancer patients after gastrectomy. Since 2014, our hospital has begun a carefully tailored ERAS program for these patients. This perioperative management protocol included most of the ERAS components used by many institutes^14,21–24^. After the surgery is completed, early oral feeding is then included in the program,and the comparative results in various aspects are reported below.

## Materials and Methods

### Study design and inclusion/exclusion criteria

From May 2014 to May 2018, a total of 87 patients with gastric cancer who underwent selective gastrectomy were enrolled. Inclusion criteria included gastric carcinoma by endoscopic biopsy, ages 18 to 80 years, selective gastrectomy (subtotal or total resection; by open or laparoscopic approach), normal organ function, no history of chemotherapy or radiotherapy before surgery. Exclusion criteria included cancer patients with obstruction, perforation or bleeding, patients with another cancer simultaneously, patients who had previously undergone gastric surgery, and patients with impaired heart, liver, or kidney function.

### Perioperative care and follow-up

The perioperative ERAS protocols were administered to all patients according to the ERAS Society guidelines (Table 1) and the completion status of each ERAS element per participated patient was monitored and recorded. Based on previous studies^9,25,26^, patients were started on sips of water on the first postoperative day (POD). If there is no obvious gastrointestinal discomfort, such as repeated nausea, vomiting, persistent cramping pain, then give a clear liquid diet (six times a day) to patients on POD 2, then take a liquid diet on POD 3, and take a semi-liquid diet from POD 4 until hospital discharge (Table 2). Tables 3–6 gives adetailed dietary plan and calorie intake for this early oral feeding schedule. According to routine recommendations, the patient’s first day of calorie supply is about 20 kcal/kg, and about 30 kcal/kg from the second day after surgery. A joint parental nutrition program was also implemented to supplement the patient’s calorie needs as to the total calories needed per day. During the first four PODs all patients received intravenous fluids in the form of a fluid restriction regimen (Table 2). Starting on POD1, they were also encouraged to ambulate actively. Once the following objective criteria are met, all patients should be discharged on POD 5 or 6: no signs of postoperative symptoms, can ambulate without help, can take ≧60% of a meal, pain can be tolerated on oral analgesia, and a self-willingness to go home.

**Table 1:** ERAS protocol used in our department.

1. Preoperative consultation and patient’s qualification survey
2. Preoperative carbohydrate loading (800 ml of Maltodextrin drink was given 2–12 h prior to surgery)
3. Antibiotic prophylaxis (1.5 g Cefuroxime was given 30 min prior to surgery by iv)
4. Operation approach (laparoscopic gastrectomy is preferred to open gastrectomy)
5. Balanced intravenous fluid therapy (<2500 ml intravenous fluid during the day of surgery, postoperative rehydration includes intravenous infusion of amino acid and fat emulsion solution)
6. No routine gastric tube or pull the gastric tube as soon as possible after surgery
7. TAP block and standard anesthesia protocol
8. Postoperative analgesia avoiding opioids (Parecoxib sodium 40 mg Bid. iv)
9. Postoperative oxygenation therapy (4–6 L/min)
10. Postoperative antiemetic, tropisetron hydrochloride injection (4 mg, iv, qd.) when needed
11. Early oral feeding (initiate on POD 1)
12. Urinary catheter removal on POD 1
13. Abdominal drainage tube removal on POD 4
14. Full ambulation on POD 1 (walking along the corridor, at least 2 hours out of bed)

TAP, Transversus abdominis plane; POD, postoperative day; IV, intravenous infusion; qd, four times a day; Bid, twice a day.

**Table 2:** Advanced schedules of oral intake and intravenous fluid infusion (standard = 60 kg adult).

POD	Oral intake (amount)	Intravenous fluid (ml)	Total calories (kcal)
1	Water (300 ml)	2250	1150
2	Clear liquid diet (500 ml)	2000	1750
3	Liquid diet (600 ml)	1500	1800
4	Semi-liquid diet (half amount)	1000	1800
5	Semi-liquid diet (full amount)	500	1800

Note: Oral diet were supplemented with parenteral nutrition up to the calculated total calories.

**Table 3:** Early oral feeding schedule after gastric cancer surgery- Clear fluid diet on POD 2.

Meal #	Time	Components (Calories)
1	7:00 am	100 ml of 25% Maltodextrin (50 kcal)
2	9:30 am	15 g of Lotus root starch and 15 g of albumen powder in 50 ml (109 kcal)
3	11:30 am	22 g of Ensure or 23 g of Glucema in 100 ml (100 kcal)
4	14:30 pm	Rice-water plus 15 g of albumen powder in 50 ml
5	17:00 pm	22 g of Ensure or 23 g of Glucema in 100 ml (100 Kcal)
6	20:00 pm	25 g of Whey protein powder in 100 ml (100 kcal)

Note: This clear fluid diet contains 600 Kcal, 40 grams of protein and 500 ml volume in total; Ensure is from Abbott Laboratories B.V., GLUCERNA SR is also from Abbott Laboratories S.A.

**Table 4:** Early oral feeding schedule after gastric cancer surgery- liquid diet on POD 3.

Meal #	Time	Components (Calories)
1	7:00 am	100 ml of 25% Maltodextrin (50 kcal)
2	9:30 am	30 g of Lotus root starch and 25 g of whey protein powder in 100 ml (110 kcal)
3	11:30 am	22 g of Ensure or 23 g of Glucema in 100 ml (100 kcal)
4	14:30 pm	Rice-water plus 25 g of whey protein powder in 100 ml (100 Kcal)
5	17:00 pm	22 g of Ensure or 23 g of Glucema in 100 ml (100 Kcal)
6	20:00 pm	25 g of Whey protein powder in 100 ml (100 kcal)

Note: This liquid diet contains 800 Kcal, 50 grams of protein and 600 ml volume in total.

**Table 5:** Early oral feeding schedule after gastric cancer surgery- A semi liquid diet (half quantity) on POD 4.

Meal #	Time	Components (Calories)
1	7:00 am	White porridge (25 g of glutinous rice) and 30 g of fleshy pine
2	9:30 am	33 g of Ensure or 35 g of Glucema in 150 ml (150 kcal)
3	11:30 am	Minced meat porridge (25 g of glutinous rice, 50 g of green vegetables 50 g, 25 g of pork leg meat)
4	14:30 pm	33 g of Ensure or 35 g of Glucema in 150 ml (150 kcal)
5	17:00 pm	Minced meat noodles (25 g of noodles, 50 g of Chinese cabbage, 25 g of pork leg meat)
6	20:00 pm	25 g of Whey protein powder in 150 ml (100 kcal)

Note: This liquid diet contains 878 Kcal, 47.5 grams of protein in total.

**Table 6:** Early oral feeding schedule after gastric cancer surgery- Semi-liquid diet on POD 5.

Meal #	Time	Components (Calories)
1	7:00 am	White porridge (50 g of glutinous rice) and fleshy pine 30 g
2	9:30 am	55 g of Ensure or 58 g of Glucema in 200 ml (250 kcal)
3	11:30 am	Minced meat porridge (50 g of glutinous rice, 100 g of green vegetables, 50 g of pork leg meat)
4	14:30 pm	55 g of Ensure or 58 g of Glucema in 200 ml (250 kcal)
5	17:00 pm	Minced meat noodles (50 g of noodles, 100 g of Chinese cabbage, 50 g of pork leg meat)
6	20:00 pm	25 g of Whey protein powder in 100 ml (100 kcal)

Note: This semi-liquid diet contains 1442 Kcal, 71 grams of protein in total.

### Assessment of surgical outcomes

The primary outcomes were postoperative complication (graded according to the Clavien-Dindo classification)^27,28^, the duration of postoperative hospital stay (DPOS), the time of first flatus (gas passage), the 30-day post-discharge readmission rates and hospital mortality. The secondary outcome was the tolerance of early oral feeding (capable of ingesting 60% or more of a given meal means tolerated, otherwise it is intolerable) and adherence to other provisions of the ERAS protocol.

### Statistical analysis

Since the sample size in this study was small (n = 87), an exploratory study was performed on all patients. Descriptive statistics were calculated and in accordance with general principles. If the continuous variables are normally distributed, they are presented as mean ± sd. Otherwise, they will be displayed by median (interquartile range; IQR) values and compared by a non-parametric test (Mann-Whitney), while the count and frequency are used for categorical variables. To explore the relationship between the possible influencing factors and the likehood of a responsive event—Early Oral Feeding Intolerance, univariate Logistic regression analysis was performed firstly and followed by a stepwise regression method. OR (95% CI) is calculated for each predictor, and OR > 1 indicates a high risk of intolerance, and OR < 1 indicates a low risk of intolerance. SPSS ver. 22.0 (SPSS Inc., Chicago, IL, USA) was utilized throughout, and statistical significance was accepted for P-value of < 0.05.

### Ethical approval

This study was approved by the local Ethics Review Committee of Tongren Hospital, Shanghai Jiao Tong University School of Medicine. The study was carried out in accordance with the Declaration of Helsinki in 1964 and its later amendments. All procedures performed in studies involving human participants were in accordance with the ethical standards of the institutional and/or national research committee and are consistent with the Helsinki declaration of 1964 and its subsequent amendments or similar ethical standards.

Informed consent was obtained from all individual participants included in the study.

## Results

### Clinicopathological characteristics

From May 2014 to May 2018, 87 eligible patients were enrolled in this retrospective study. Table 7 summarizes the clinicopathological features of all participants. There were 56 males and 31 females. Among them, 23 (26%) are over 70 years old. 39 (43%) had one or more preoperative medical conditions, 52 (60%) had a body mass index (BMI) of less than 25 kg/m^2^, and 84 (97%) were considered as having American Society of Anesthesiologists (ASA) grade I or II. 69 patients (79%) underwent subtotal gastric resection, and 18 patients (21%) patients underwent total gastrectomy; a total of 54 patients (62%) underwent laparoscopic surgeries, with 7 and 47 cases of total and subtotal gastric resections, respectively. According to the 7th edition of the AJCC TNM classification, there are 28 (32%), 11 (13%), 46 (53%) and 2 (2%) in TNM stage I, II, III and IV respectively.

**Table 7:** Clinicopathological features.

	Patients (N = 87)
Age	
<70	64 (73%)
≥70	23 (26%)
Sex	
Male	56 (64%)
Female	31 (36%)
Medical comorbidity	
None	50 (58%)
One	27 (31%)
Two or more	10 (12%)
ASA grade	
I	47 (54%)
II	37 (43%)
II	3 (3%)
BMI	
<25 kg/m^2^	52 (60%)
≥25 kg/m^2^	35 (40%)
Resection type	
Total gastrectomy	18 (21%)
Subtotal gastrectomy	69 (79%)
Operative approach	
Open	33 (38%)
Laparoscopic	54 (62%)
TNM stage	
I	28 (32%)
II	11 (13%)
III	46 (53%)
IV	2 (2%)

BMI, body mass index; ASA, American Society of Anesthesiologists.

### Surgical outcomes

According to Table 8, the median time (IQR) to the first flatus was 2 (1) days. After meeting discharge criteria, 59 patients (68%) were discharged within POD 6, and the median (IQR) duration of postoperative hospital stay was 6 (3) days for all patients (Table 8).

**Table 8:** Surgical outcomes.

	Patients (N = 87)
The onset of first flatus (Median (IQR) days)	2 (1)
Early oral feeding	67 (77%)
Water intake (POD1)	85 (98%)
Clear liquid diet (POD2)	80 (92%)
Liquid diet (POD3)	71 (82%
Semi-liquid diet (POD4)	67 (77%)
Postoperative hospital stay (Median (IQR) days)	6 (3)
Rehospitalization	2 (2%)
Hospital mortality	0

POD, postoperative days; SD, standard deviation.

Postoperative complications occurred in 23 (26%) patients after surgical resection and recovery. Of these, 13 (15%) were mild (Clavien-Dindo grade 1), of which 9 (10%) had just postoperative nausea and vomiting, and 3 (3%) were severe (Clavien-Dindo grade 3–5). Table 9 shows a detailed description of these complications. Two patients were readmitted due to gastric retention and intestinal ileus within 30 days after discharge, and were cured by conservative medical treatments. No single in-hospital mortality was observed.

**Table 9:** Types of surgical complications according to Clavien-Dindo classification.

Clavien-Dindo classification		Surgical complications	Patients (N = 87)
I	15%	Surgical site infection	0
		Postoperative nausea and vomiting	9
		Postoperative paralytic ileus	3
		Fever of unknown origin	1
II	8%	Urinary tract infection	1
		Infectious diarrhea	1
		Pneumonia	4
		Surgical site infection (requiring antibiotics)	1
IIIA	0 3%	Anastomotic leakage (managed endoscopically)	0
IIIB		Anastomotic leakage (reoperation)	1
		Intraperitoneal hematoma	0
		Postoperative bleeding	2
IV	0	Anastomotic leakage (ICU stay)	0
V	0	Death	0

### The tolerability to early oral feeding

**Figure 1:**
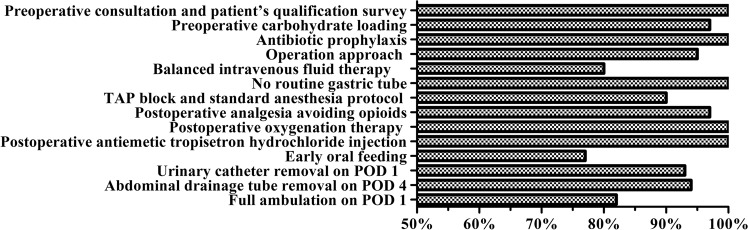
Compliance with ERAS protocol elements.

Sixty-seven patients (77%) successfully started water intake on POD 1 and successfully started a half-amount semi-liquid diet on POD 4 (Table 8). Twenty (23%) patients were unable to tolerate the postoperative early oral feeding schedule. Of these 20 patients, 14 patients delayed the oral feeding schedule due to gastrointestinal symptoms, such as recurrent nausea, vomiting, and persistent abdominal discomfort or pain, and 6 patients were discontinued on oral intake completely due to postoperative complications (3 cases of paralytic ileus, 2 cases of luminal bleedings, and 1 case of anastomotic leakage) (Table 9). In addition, according to Fig. 1, it can be seen that in all items of the ERAS protocol, patients had the lowest compliance with early oral feeding (77%), followed by the other two lower: Balanced intravenous fluid therapy (80%) and Full ambulation on POD 1 (82%).

### Possible predictors of early oral feeding intolerance after gastric resection

Twenty (23%) patients failed to follow the early postoperative oral feeding schedule after the start of water intake or semi-liquid diet. To reveal variables that indicate early postoperative oral feeding intolerance, we compared success and failure in terms of patient’s age, sex, medical comorbidity, ASA grade, BMI, resection type, operative approach, and TNM stage. Results of the univariate logistic regression analysis (Table 10) demonstrate that the operative approach may be a relevant factor indicating patients’ low compliance with early oral feeding schedule in the ERAS protocol, and it is the only factor in the model that uses a stepwise approach. The TNM stage III *vs*. I (P = 0.06) also showed a marginal significance associated with the schedule deviation.

**Table 10:** Relationship between potential factors and early oral feeding intolerance -univariate logistic regression analysis.

Variables	Level	N	Odds Ratio (95% CI)	OR P-value
Age	<70	64		
	≥70	23	1.26 (0.42–3.80)	0.681
Sex	Male	56	—	—
	Female	31	0.72 (0.25–2.11)	0.55
Medical comorbidity	None	50	—	—
	One	27	1.24 (0.42–3.69)	0.698
	Two or more	10	0.89 (0.16–4.79)	0.889
ASA grade	I	47		
	II	37	0.76 (0.26–2.21)	0.619
	III	3	6.55 (0.54–79.23)	0.14
BMI	<25 kg/m2	52		
	≥25 kg/m2	35	0.41 (0.13–1.26)	0.12
Gastrectomy type	Subtotal gastrectomy	69	0.36 (0.12–1.12)	0.079
	Total gastrectomy	18	—	—
Operative approach	Open	33		
	Laparoscopic	54	0.30 (0.11–0.85)	0.024**
TNM stage	I	28		
	II	11	1.85 (0.26–12.94)	0.535
	III	46	3.65 (0.94–14.09)	0.061
	IV	2	8.33 (0.41–170.64)	0.169

CI, confidence interval; OR, odds ratio; if the p-value is less than 0.05, it is in bold and flagged with two stars (**).

## Discussion

Gastric cancer is one of the most common malignancies, and resection is the only way to prolong the survival of patients with gastric cancer^1,29^. In recent years, with the increasing diagnosis rate of early gastric cancer, the continuous improvement of laparoscopic gastrectomy, the overall survival rate of patients after gastric cancer surgery is also increased^30–34^. Therefore, how to improve the quality of postoperative life and faster recovery of patients with gastric cancer has become the focus of medical workers.

Preoperative care programs based on the ERAS protocol have been proposed to maintain physiological function and accelerate recovery after various types of surgery^7,14,18^. Dietary care is an important factor affecting postoperative recovery in patients with gastric cancer^7,8^. In the past, patients who underwent gastric cancer resection were routinely fasted for 3 to 4 days, and gastrointestinal decompression and enteral feeding were performed through the nasogastric tube until the patient’s intestinal function recovered. However, in recent years, early postoperative oral feeding as an important component of accelerated rehabilitation surgery has been extensively used in clinical practice abroad, especially in colorectal surgery^18,20,35,36^. A growing body of clinical evidence suggests that early oral feeding in patients with colorectal cancer is safe and tolerable. Compared with traditional postoperative diet, oral feeding within 24 hours after colorectal surgery can speed up the recovery of intestinal function and promote early recovery of patients without increasing postoperative complications. However, compared with colorectal cancer, the safety and reliability of early oral feeding after gastric cancer surgery has been lacking high-level therapeutic evidence. Therefore, this method has not been adopted in gastric surgery for a long time because of fear that early food intake may cause anastomotic leakage due to direct stimulation of anastomotic sites and increased intraluminal pressure. Until 2004, it was reported for the first time by Suehiro *et al*. that patients undergoing gastrectomy can accelerate their recovery after postoperative early oral intake^7^. In the following decade, more and more studies have shown that early oral feeding after gastrectomy is safe and feasible^8–11^. In July 2014, the European Association of Accelerated Rehabilitation Surgery first developed and released the guide to Accelerated Rehabilitation for Gastric Resection Surgery (hereinafter referred to as the Guide), which states that patients who underwent total gastrectomy can be administered the postoperative oral diet according to their tolerance as of POD 1^13^.

In this case,due to the lack of similar studies in China, we conducted a retrospective study to assess the safety and efficacy of early oral feeding and proposed a versatile ERAS protocol for accelerated recovery of Chinese patients after gastric surgery. Overall, this study provides satisfactory short–term clinical outcomes and we strongly believe that patients undergoing gastrectomy should be encouraged to begin the early oral feeding carefully and adjust the feeding schedule based on their tolerability. In our study, the primary outcome was a postoperative complication rate of 26%, and a severe 3% (Clavien-Dindo 3–5). It is comparable to the rates in other reports^8,11,37,38^. It is worth noting that Clavien-Dindo classification of surgical complications can compensate for other reports that are graded according to different criteria. These reports only identify severe complications, and often ignore complications that do not require intervention^27^. In addition, in our patient group, 68% of patients were discharged on POD 6 and all patients had a median postoperative hospital stay (IQR) of 6 (3) days. Similarly, according to the study by Hur and Colleagues, that was 8.03 ± 1.43 days in the early oral feeding group and 9.9 ± 2 days in the control group^39^. In addition, Jeong and colleagues reported that in their study, hospital stay was 7.4 days in the early feeding group and 8.9 days in the control group^11^. Although there is no comparison done between the early oral feeding group and a control group in our study, the duration of postoperative stay was shortened compared to the results from the above-mentioned two studies and other studies as well^21,37,38^. All of these results indicate that patients who underwent gastrectomy had shorter postoperative hospital stays without increasing postoperative complications^11^.

Although many studies have applied early oral feeding for patients after gastric surgery, and reported improvements in postoperative outcomes and accelerated recovery, the actual implementation of early oral feeding varied from one to the other. To date, there is still no consensus on appropriatetiming, composition, frequency and volume or amount of early oral feeding. We performed an early oral feeding of 204 patients undergoing colorectal surgey (adjusted according to the dietary habits of local Chinese residents), and more than 90% of them were tolerated (unpublished data). Our previous data also found that patients who underwent gastrectomy were less tolerant to oral feeding than patients undergoing colorectal surgery. Based on our past experience with early oral feeding programs for patients undergoing colorectal surgery, combined with other early oral feeding schedules found in recent studies, we developed a stepwise, Chinese patient-specific early oral feeding plan from water to other liquids, followed by semi-fluids, and finally have a normal diet. We initiated the oral intake at the target volume within 24 hours of surgery. According to our findings, 77% of the patients were able to adhere to the oral feeding schedule without causing any adverse events, and this is moderate in the tolerability (57–93%) after gastric cancer surgery reported in previous studies^10,11,21,38^. In addition, according to the dietary habits of our local patients, we recommend the following dietary guidelines for discharged patients: from the discharge time to 3 weeks after surgery, eat 6 to 8 semi-liquid diets a day, and each time the calorie is 200~250 kcal; 3~4 weeks after the operation, gradually transit to a normal diet, eat 6 times a day with the calorie of each meal between 250 and 300 kcal.

In theory, it is assumed that the age of the patient, the extent of tumor resection, the surgical approach, and the stage of the tumor allaffect early oral feeding tolerance in postoperative gastric cancer patients. For instance, a recent study by Shimizu *et al*. supported that compared to the distal gastrectomy (DG) groups, the postoperative stay was significantly shorter in the total gastrectomy (TG) groups, and the incidence of postoperative complications after early oral feeding supplementation was lower^40^. However, no differences were found in the duration of postoperative stay between early oral feeding and normal groups of the patients receiving distal gastrectomy (DG). But a high incidence of postoperative complications was found in the early oral feeding groups of patients with DG^40^. This report seems to indicate that the extent of resection (rather than surgical approach) may shorten the postoperative stay and reduce postoperative complications as beneficial results of early oral feeding. Nevertheless, as the authors say, the above conclusions are far from confirmed due to insufficient sample size in the TG group, which requires further researches.

Interestingly, our results demonstrated that laparoscopic gastrectomy can reduce the chance of early oral feeding intolerability compared with patients undergoing an open operation, with an Odds Ratio of 0.30. However, due to the small sample size this estimated OR is not precise enough, reflected by a much wider range of 95% CI (0.11–0.85). Also because of that, we realized that there is no point in conducting a further stratified comparison analysis between open or laparoscopic operation groups because of the limited number of cases of sub- or total gastric resection within each group. Therefore, caution should be exercised when expanding this conclusion. A complete model with all potential factors considered was fitted but it failed to reliably estimate the parameters and thus it won’t be presented in this paper. We propose to conduct a multi-center case-control study with a much larger sample size in the future and further corroborate our findings. In addition, it is worth pointing out that both postoperative fluid restriction regimen and postoperative mobilization protocol may also affect early oral feeding. Thus, it is necessary to conduct further research on this to better address the above-discussed factors affecting early feeding tolerance before we can improve our early oral feeding schedule to better promote the rehabilitation of patients. Additional analysis of the immunological functions of postoperative gastric cancer patients will also be needed to optimize the early oral feeding schedules.

## Conclusion

In conclusion, this retrospective study concluded that our ERAS protocol incorporating a stepwise, Chinese patient-specific early oral feeding schedule was also safe and beneficial compared to similar overseas studies in the past. It can shorten the postoperative hospital stays without increasing the incidence of postoperative complications. In addition, as far as we know, this is the first domestical report on detailed and most importantly suitable early feeding plan for Chinese patients after gastric surgery. Therefore, although the study has some limitations, such as small sample size and lack of control studies, it still deserves further research and promotion. Finally, we recommend that a customized, optimized oral feeding plan should be developed for local Chinese patients with their dietary habits, races, and other factors considered. Together with fine-tuning of other essential elements of the current ERAS protocol, it is foreseeable that a more effective ERAS protocol will be offered in the near future, includinging an optimized early oral feeding schedule for gastric cancer surgery.

## Data Availability

The datasets generated during and/or analyzed during the current study are available from the corresponding author upon reasonable requests.
